# Structural Data on the Periplasmic Aldehyde Oxidoreductase PaoABC from *Escherichia coli*: SAXS and Preliminary X-ray Crystallography Analysis

**DOI:** 10.3390/ijms15022223

**Published:** 2014-01-31

**Authors:** Ana Rita Otrelo-Cardoso, Márcia Alexandra da Silva Correia, Viola Schwuchow, Dmitri I. Svergun, Maria João Romão, Silke Leimkühler, Teresa Santos-Silva

**Affiliations:** 1 REQUIMTE, Departamento de Química, Faculdade de Ciências e Tecnologia, Universidade Nova de Lisboa, Caparica 2829-516, Portugal; E-Mails: a.cardoso@campus.fct.unl.pt (A.R.O.-C.); marcia.correia@fct.unl.pt (M.A.S.C.); mjr@fct.unl.pt (M.J.R.); 2 Institut für Biochemie and Biologie, Universität Potsdam, Karl-Liebknecht-Str. 24-25, Golm, Potsdam 14476, Germany; E-Mails: visch@uni-potsdam.de (V.S.); sleim@uni-potsdam.de (S.L.); 3 European Molecular Biology Laboratory, Hamburg Outstation, Notkestrasse 85, Hamburg 22607, Germany; E-Mail: svergun@embl-hamburg.de

**Keywords:** periplasmic aldehyde oxidoreductase, X-ray crystallography, small angle X-ray scattering, crystal twinning

## Abstract

The periplasmic aldehyde oxidoreductase PaoABC from *Escherichia coli* is a molybdenum enzyme involved in detoxification of aldehydes in the cell. It is an example of an αβγ heterotrimeric enzyme of the xanthine oxidase family of enzymes which does not dimerize via its molybdenum cofactor binding domain. In order to structurally characterize PaoABC, X-ray crystallography and small angle X-ray scattering (SAXS) have been carried out. The protein crystallizes in the presence of 20% (*w*/*v*) polyethylene glycol 3350 using the hanging-drop vapour diffusion method. Although crystals were initially twinned, several experiments were done to overcome twinning and lowering the crystallization temperature (293 K to 277 K) was the solution to the problem. The non-twinned crystals used to solve the structure diffract X-rays to beyond 1.80 Å and belong to the C2 space group, with cell parameters *a* = 109.42 Å, *b* = 78.08 Å, *c* = 151.77 Å, β = 99.77°, and one molecule in the asymmetric unit. A molecular replacement solution was found for each subunit separately, using several proteins as search models. SAXS data of PaoABC were also collected showing that, in solution, the protein is also an αβγ heterotrimer.

## 1. Introduction

Molybdenum is a transition metal that is incorporated, as a biologically active cofactor (molybdenum cofactor, Moco), in a class of widely distributed proteins collectively known as molybdoenzymes [1]. Moco is associated with a wide range of redox enzymes and is found in most organisms from bacteria to humans. The metal in Moco is coordinated to a pterin derivative called molybdopterin to form the molybdenum containing molybdopterin (Mo-MPT) cofactor [2]. A wide variety of transformations are catalyzed by these enzymes at carbon, sulfur and nitrogen atoms, which include the transfer of an oxo group or two electrons to or from the substrate. Depending on the ligands of the molybdenum center in Moco, molybdoenzymes are classified into three families: (*i*) the xanthine oxidase (XO) family, characterized by a cyanolyzable equatorial sulfur ligand coordinated to the molybdenum atom; (*ii*) the sulfite oxidase family, with two oxo ligands at the molybdenum center; and (*iii*) the dimethylsulfoxide (DMSO) reductase family, where one molybdenum atom is coordinated by two dithiolene groups [1,3,4]. While in eukaryotes Moco is present solely in its Mo-MPT form, in bacteria additional variability of the Moco is achieved by attachment of a second nucleotide, GMP or CMP to the phosphate group of MPT [5–7].

The XO family of molybdoenzymes comprises a number of different enzymes in prokaryotes and eukaryotes, all transferring oxygen derived from water to their substrate. Most enzymes of the XO family are well characterized as purine and/or aldehyde oxidizing enzymes with broad substrate specificities, but also several more specific enzymes like carbon monoxide dehydrogenase and nicotine dehydrogenase have been described [8,9]. Well-characterized enzymes with aldehyde-oxidizing activity are *Desulfovibrio gigas* aldehyde oxidoreductase and mammalian aldehyde oxidases [10]. Mammalian aldehyde oxidases are expressed at high levels in the liver and in the lung and have been implicated in the detoxification of environmental pollutants and xenobiotics [11]. Bacterial aldehyde oxidases and aldehyde dehydrogenases were identified in different bacteria, including *Methylococcus* sp., *Pseudomonas* sp., *Streptomyces moderatus* [12], *Amycolatopsis methanolica* [13] and *Pseudomonas testosteroni* [14]. In addition, xanthine dehydrogenases (XDH) capable of oxidizing various purine and aldehyde substrates were characterized in bacteria like *Rhodobacter capsulatus* [15], *Pseudomonas putida* 86 [16,17], and *Veillonella atypica* [18,19]. With the exception of *R. capsulatus* and *Pseudomonas aeruginosa* XDH [20,21] which binds Mo-MPT, all bacterial XDHs characterized to date bind the molybdopterin cytosine dinucleotide (MCD) form of Moco. In these XDHs, molecular masses range from 140 to 300 kDa and different subunit structures were observed, like α_2_ in *Steptomyces cyanogenus* [22], αβγ in *V. atypica* [19], α_3_ in *P. putida* [23], α_2_β_2_ in *R. capsulatus* [15], α_2_β_2_ in *Comamonas acidovorans* [24,25], and α_4_β_4_ in *P. putida* 86 [17]. However, in general, enzymes of the XO family possess the same overall architecture [26], with two distinct [2Fe–2S] clusters bound to the N-terminal domain or subunit, a flavin adenine dinucleotide (FAD) bound to a central domain or subunit (with the exception of *D. gigas* aldehyde oxidoreductase in which the FAD binding domain is absent [27]) and the Moco-binding domain at the *C*-terminus.

Among the members of the xanthine oxidase family in *E. coli* are the xanthine dehydrogenase XdhABC, the periplasmic aldehyde oxidoreductase PaoABC, and the so far uncharacterized xanthine dehydrogenase homologue XdhD [28]. The *paoABCD* operon encodes for a molybdenum-containing iron–sulfur flavoprotein that is located in the periplasm [29]. The 135 kDa enzyme comprises a noncovalent (αβγ) heterotrimer with a large (78.1 kDa) molybdenum cofactor (Moco)-containing PaoC subunit, a medium (33.9 kDa) FAD-containing PaoB subunit, and a small (21.0 kDa) 2× [2Fe2S]-containing PaoA subunit, which also contains the Tat-leader peptide for the localization to the periplasm. PaoD is not a subunit of the mature enzyme, and the protein is expected to be involved in Moco modification and insertion into PaoABC. Analysis of the form of Moco present in PaoABC revealed the presence of the MCD cofactor [29]. Kinetic characterization of the enzyme showed that PaoABC converts a broad spectrum of aldehydes, with a preference for aromatic aldehydes. The terminal electron acceptor of the enzyme has not been identified to date, however, NAD^+^ is not used as terminal electron acceptor and the reactivity with molecular oxygen is very slow. Complete growth inhibition of *E. coli* cells devoid of genes from the *paoABC* operon was observed by the addition of cinnamaldehyde to a low-pH medium. This finding showed that PaoABC might have a role in the detoxification of aromatic aldehydes for *E. coli* under certain growth conditions [29].

Here, we describe the crystallization experiments and preliminary X-ray diffraction data of the enzyme purified after homologous expression in *E. coli*. Additional SAXS experiments confirmed the αβγ heterotrimeric structure of the enzyme.

## 2. Results and Discussion

### 2.1. Crystallization and Data Processing

**Figure 1.**
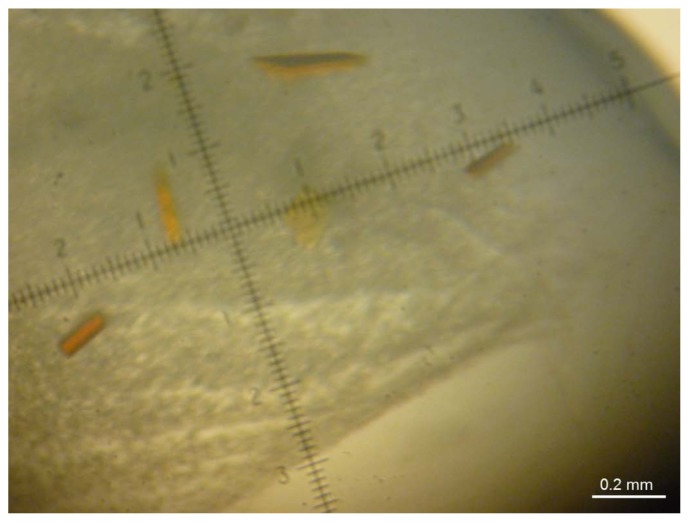
Crystals of PaoABC grow in 0.2 M ammonium iodide and 22% (*w*/*v*) polyethylene glycol 3350.

To crystallize the periplasmic aldehyde oxidoreductase (PaoABC) from *E. coli* several screens were tested. The protein crystallizes with 0.2 M ammonium iodide and 20% (*w*/*v*) polyethylene glycol 3350 (Figure 1). Two crystallization temperatures were used, 277 and 293 K (crystals_A and crystals_B, respectively), and several plate-like crystals appeared after four or two days, respectively. The datasets collected for both types of crystals diffracted to high resolution (1.67 and 1.8 Å, respectively) and presented similar processing statistics (Table 1). The crystals belong to the centred monoclinic space group C2 and the Matthews coefficient calculations suggest the presence of one heterotrimer (αβγ) per asymmetric unit, (Matthews coefficient of 2.7 Å^3^/Da) with a solvent content of 48%.

Analysis using TRUNCATE [30] and XTRIAGE [31] showed normalized structure amplitudes <E> of 0.903 and 0.936, for the crystals grown at 277 K and 293K, respectively. Since the expected value for a non-twinned dataset is 0.886 and for a perfectly twinned data set is 0.94, the obtained values suggest the presence of twinning for the crystals prepared at highest temperature. As several problems may arise during phasing and refinement [32] for twinned data, the non-twinned dataset was used to solve the PaoABC structure.

### 2.2. Structure Determination

To solve the structure of PaoABC, sequence alignment searches were performed separately for each subunit in order to find the best homologous models that could lead to good initial phases obtained by molecular replacement. For subunit A, 4-hydroxybenzoyl-CoA reductase from *Thauera aromatica* (*Ta*HBRC, PDB code 1rm6) [33]), quinoline 2-oxidoreductase from *Pseudomonas Putida* 86 (*Pp*QoR, PDB code 1t3q) [34] and carbon monoxide dehydrogenase from *Hydrogenophaga pseudoflava* (*Hp*CODH, PDB code 1ffu) were used; for subunit B, *Ta*HBCR, *Hp*CODH (PDB code 1ffu and 1ffv) were used; and for subunit C, *Ta*HBCR, *Bos taurus* xanthine oxidase complexed with xanthine (B*t*XO, PDB code 3eub) and reduced *Bt*XO in complex with arsenite (PDB code 3sr6) [35] were used as models.

In order to optimize the structure solution, Chainsaw [36] was used, pruning non-conserved residues prior to PHASER [37], from the CCP4 package. Three steps of molecular replacement were necessary to solve the structure of the protein, finding the position of each subunit separately. In the final solution, positive electron density was found at the expected position of the cofactors MCD, 2× [2Fe-2S] clusters and FAD, that had been excluded from the search models, thus confirming the correctness of the solution. Anomalous maps have also been calculated in order to help positioning heavy atoms in the electron density. Even though molybdenum and iron have low anomalous contribution at 0.976 Å wavelength (distant from the adsorption peak at 0.62 Å and 1.74 Å, respectively), strong anomalous peaks could be found for the Mo atom at the enzyme active site and for iron at the electron transfer centres.

The data-collection and processing statistics are presented in Table 1 and the crystal structure is currently under refinement.

### 2.3. Quaternary Structure Analysis of PaoABC

**Figure 2.**
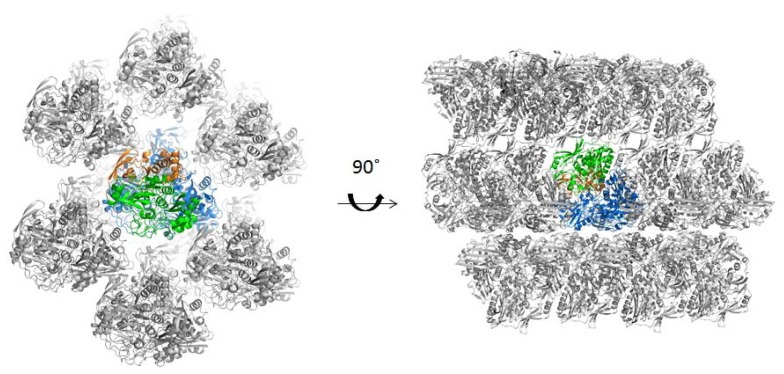
Crystal packing of PaoABC. Cartoon representation of several asymmetric units of PaoABC untwinned crystals with subunit A in orange, B in green and C in blue when viewed along the c axis and rotated 90°. Picture prepared using PyMOL 1.5.0.3 [40].

While all the enzymes of the XO family that have so far been analysed by crystallography exhibit dimeric structures (homodimers or dimers of heterotrimers (heterodimers) [38]), biochemical analysis suggested that PaoABC does not dimerize via its Moco-binding domain and remains an αβγ heterotrimer in solution. Although only one molecule is found in the asymmetric unit, we analysed the crystal packing from the position of the crystallographic dyads. This was first done by visual inspection and no tight packing could be identified (Figure 2). In addition, we used the PISA server (Protein Interfaces, Surfaces and Assemblies) [39] that predicted the ABC arrangement based on chemical thermodynamic calculations. The solvation free energy gain (Δ*G*^int^) of −163.1 kcal/mol and the free energy of assembly dissociation (Δ*G*^diss^) of 22.8 kcal/mol shows that PaoABC is thermodynamically stable as αβγ heterotrimer [39].

### 2.4. Small-Angle X-ray Scattering of PaoABC

**Figure 3.**
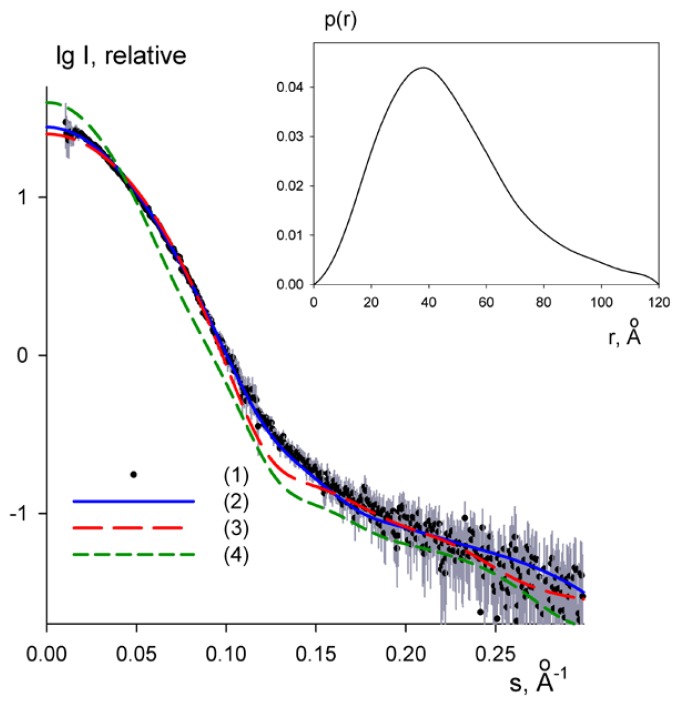
SAXS data from PaoABC in solution. The experimental data (**1**) are displayed as black dots with grey error bars, and the scattering computed from the models is shown as smooth lines: (**2**) is the scattering from the *ab initio* shape, (**3**) and (**4**) are scattering computed from the monomer and dimer of 1rm6, respectively. Logarithm of the scattering intensity is displayed as a function of the momentum transfer s. Insert, the characteristic function of PaoABC computed from the scattering data.

SAXS experiments have also been performed for PaoABC in order to further explore the structure and oligomerization state of the protein in solution. Monodisperse concentrated solutions of PaoABC were measured and the processed scattering profile of PaoABC are presented in Figure 3. The structural parameters including the radius of gyration (*R*_g_), the maximum particle dimension (*D*_max_) and the excluded (Porod) volume of the hydrated particle computed from the experimental data are summarized in Table 2. The distance distribution function, *P*(*r*), obtained from the experimental data suggests that the protein is well folded and has a globular structure (Figure 3). The molecular mass (MM) of the solute estimated from the forward scattering *I*(*0*) and also from the Porod volume (Table 2) are both close to that of the monomeric PaoABC (136 kDa) indicating that the protein is a heterotrimer in solution.

**Figure 4.**
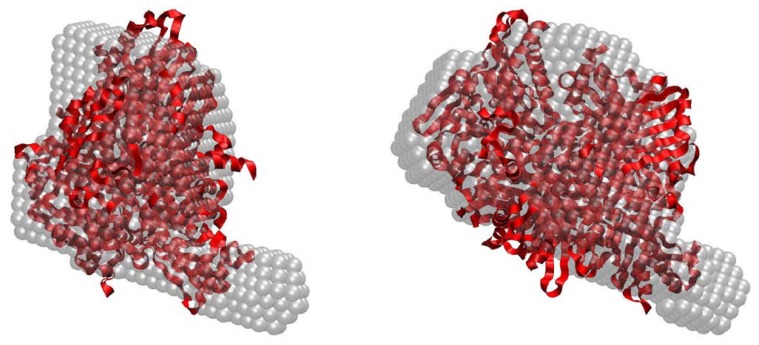
Superposition of the *ab initio* envelope of PaoABC (transparent beads, the most typical reconstruction from twenty DAMMIF runs) with the ribbon representation of the crystallographic monomer of 1rm6. Right panel is rotated 90 degrees counterclockwise along the vertical axis.

The overall shape of PaoABC was calculated *ab initio* from its scattering profile using programs DAMMIN [41] and DAMMIF [42]. The most typical model out of 20 reconstructions as analysed by DAMAVER [43] is overlapped in Figure 4 with the crystallographic structure of the monomer of *Ta*HBRC (PDB code 1rm6, [33]). The reconstructed shape matches well with the overall appearance of the crystal structure. The experimental SAXS data were also fitted by the scattering profile calculated from the crystal structure of the monomeric 4-hydroxybenzoyl-Coa reductase using CRYSOL [44]. The crystal structure yielded *R*_g_ = 31 Å, somewhat smaller than the experimental value. The fit showed a discrepancy of χ = 1.3 (Figure 3) confirming that the overall shape of PaoABC is reasonably close to that of monomeric 1rm6, but also showing systematic deviations and indicating that PaoABC may be somewhat more extended in solution. In contrast, the scattering computed from the crystallographic dimer of 1rm6 provides an extremely poor fit to the SAXS data (Figure 3) with discrepancy χ = 3.1 and also the radius of gyration of the dimer (*R*_g_ = 43 Å) does not match the experimental data. Taken together, the SAXS data indicate that PaoABC is a heterotrimeric protein in solution with the overall structure similar but somewhat more extended than that of the monomeric 4-hydroxybenzoyl-Coa reductase.

## 3. Experimental Section

### 3.1. Crystallization

PaoABC was expressed and purified using the procedure described previously [29]. The enzyme was concentrated to 20 mg/mL in 50 mM Tris-HCl pH 7.5, 250 mM NaCl, 1 mM EDTA with a Vivaspin 20 ultrafiltration device (Sartorius Stedim Biotech S.A., Goettingen, Germany). The final concentration was determined from the absorbance at 445 nm, using an extinction coefficient of 23,686 M^−1^·cm^−1^ for the native enzyme. The extinction coefficient was determined on the basis of FAD content after trichloroacetic acid precipitation [45].

The first crystallization screening experiments were performed at 293 K by hanging-drop vapour diffusion with 1 μL of protein to 1 μL of precipitant solution on 24 well crystallization plates (Molecular Dimensions, Suffolk, UK) using several commercial screenings, namely PEG/Ion HT (Hampton Research, Aliso Viejo, CA, USA), JBScreen Classic 1–10 (Jena Bioscience, Jena, Germany), and an 80 condition in-house screen (based on the screen of Jancarik *et al*. [46]).

PaoABC crystallized only in one condition of the commercial screen PEG/Ion HT that contains 0.2 M ammonium iodide and 20% (*w*/*v*) polyethylene glycol (PEG) 3350. Red, plate shape crystals appeared within two days. However, the first datasets (collected and processed) revealed that the crystals measured were twinned, with a twinning fraction of 50% [31]. To overcome this, several concentrations of ammonium iodide (between 0.1 M and 0.25 M), percentage of PEG 3350 (between 10% and 30%), proportions of drop and additives (Additive 1 and 2, Hampton Research) were tested but without success. When the crystallization temperature was changed from 293 K to 277 K, the crystals took four days to appear reaching maximum dimensions of 0.1 × 0.1 × 0.02 mm^3^ in the same crystallization condition. These crystals were untwinned and were used to solve the structure.

### 3.2. Data Collection and Processing

The crystals were flash-cooled directly in liquid nitrogen using as cryoprotectant a solution of 30% (*v*/*v*) glycerol, 0.2 M ammonium iodide and 22% (*w*/*v*) polyethylene glycol 3350 and maintained at 100 K under a stream of nitrogen gas during data collection.

For the crystals prepared at 293 K, several datasets were collected at beamline ID14-1 and BM14 at the European Synchrotron Radiation Facility (ESRF, Grenoble, France) (twinned crystals—A). For the crystals that were prepared at 277 K, the data was collected at X06DA—PXIII at Swiss Light Source (SLS, Villigen, Switzerland) (non-twinned crystals—B). Crystals A and B diffracted up to 1.67 Å at a wavelength of 0.934 Å and 1.80 Å at a wavelength of 0.976 Å, respectively. Both crystal types belong to C2 space group with similar unit-cell parameters: *a* = 109.50, *b* = 78.16, *c* = 151.84 Å, β = 100.15° for crystals A and *a* = 109.42, *b* = 78.08, *c* = 151.77 Å, β = 99.77° for crystals B. Matthews coefficient was calculated (*ca* 2.7 Å^3^/Da) [47] suggesting the presence of one heterotrimer per asymmetric unit, with a solvent content of 48%.

Data were processed with iMOSFLM v.1.0.7 [48] and SCALA [49] from the CCP4 program package v. 6.3.0 (Collaborative Computational Project, Number 4, 1994) [50].

### 3.3. Structure Solution

Structure determination was performed with PHASER [37] using as molecular models the 4-hydroxybenzoyl-CoA reductase from *Thauera aromática* (*Ta*HBRC, PDB code 1rm6) [33], Quinoline 2-Oxidoreductase from *Pseudomonas Putida* 86 (*Pp*QoR, PDB code 1t3q) [34] and Carbon monoxide dehydrogenase from *Hydrogenophaga pseudoflava* (*Hp*CODH, PDB code 1ffu) [51] for subunit A; *Ta*HBCR, hpCODH (PDB code 1ffu and 1ffv) for subunit B and, *Ta*HBCR, Desulfo-Xanthine Oxidase with xanthine (PDB code 3eub) [52] and reduced Xanthine Oxidase in complex with arsenite (PDB code 3sr6) [35] from *Bos taurus* for subunit C. Density modification protocols [53] were applied giving initial phases with *ca* 0.7 mean figure of merit.

### 3.4. SAXS Assays

SAXS data were collected at the EMBL beamline X33 at DESY in Hamburg [54]. The measurements were performed at 293 K and different concentrations ranging from 0.3 to 30 mg/mL were used. Data were recorded using a Pilatus 1 M pixel detector at a sample-detector distance of 2.7 m and a wavelength of 1.5 Å, covering the range of momentum transfer 0.01 < *q* < 0.6 Å^−1^ (here, *q* = 4π sinθ/λ, where 2θ is the scattering angle). Sample solutions were circulated in a thermostated cuvette, positioned within a vacuum chamber. Eight frames of 15 s each were collected, normalized to the transmitted intensity, and subsequently averaged using AUTOSUB [55]. The data were processed with the ATSAS package [56] using standard procedures, corrected for buffer contribution, and extrapolated to infinite dilution using the program PRIMUS [57]. The forward scattering *I*(0) and the radii of gyration *R*_g_ were evaluated using the Guinier approximation assuming that at very small angles (*q* < 1.3/*R*_g_) the intensity is represented as *I*(*s*) = *I*(0) exp(− ( *qR*_g_)^2^/3). These parameters were also computed from the entire scattering pattern using the indirect transform package GNOM [58] that also provides the maximum dimension of the particle *D*_max_ and the distance distribution function *P*(*r*). The excluded volume of the hydrated particle (the Porod volume, *V*_p_) was computed using the Porod invariant [59]. The program CRYSOL was used to compute the scattering from the known high-resolution models of *Ta*HBRC (PDB code 1rm6) [44]. The *ab initio* modeling programs DAMMIN [41] and DAMMIF [42] were employed for low-resolution shape generation, and 20 models were calculated in the slow mode, using standard settings. The program DAMAVER [43] was utilized to superimpose individual structures, and to determine the averaged and the most probable reconstruction. The *ab initio* model was superimposed with the high-resolution structure of 4-hydroxybenzoyl-Coa reductase using SUPCOMB [60].

## 4. Conclusions

In this work we reported the crystallization of a periplasmic aldehyde oxidoreductase from *E. coli* using polyethylene glycol as precipitating agent. The first diffraction experiments revealed the presence of twinning in the crystals that posed difficulties in solving the structure. We could however overcome the problem by changing the crystallization temperature, which was decreased from 293 to 277 K. Using non-twinned crystals (grown at 277 K) complete data could be collected that allowed the solution of the PaoABC crystal structure by molecular replacement using the structures of related molybdenum proteins as search models. The initial calculated maps showed good electron density for the Moco, the two [2Fe-2S] and FAD. The refinement of the crystallographic model of PaoABC is currently under way and will provide valuable information regarding the residues involved in the electron pathway between the existing centres as well as details of the active site.

SAXS data have also been collected and, combined with the X-ray crystallographic information, allowed to characterize PaoABC, the first example of an enzyme of the XO family to be structurally analysed which forms a heterotrimer in solution and does not dimerize via its Moco-binding domain.

Studies of PaoABC in solution and in the crystalline state will be pursued towards the understanding of its enzymatic mechanism as well as for the clarification of the interaction of this enzyme with its cognate chaperone, the dimeric protein PaoD.

## Figures and Tables

**Table 1.** Data-collection and processing statistics for two types of PaoABC crystals. Values in parentheses are for the highest resolution shell.

	Twinned crystals (A) (293 K)	Non-twinned crystals (B) (277 K)
Data collection parameters		

X-ray Source	ID14-1 beam line (ESRF, Grenoble)	X06DA—PXIII beam line (SLS, Villigen)
Detector	ADSC Q210 CCD	PILATUS 2 M
Wavelength (Å)	0.934	0.976

Processing statistics		

Unit-cell parameters (Å, °)	*a* = 109.50	*a* = 109.42
	*b* = 78.16	*b* = 78.08
	*c* = 151.84	*c* = 151.77
	β = 100.15	β = 99.77
Space group	C2	C2
Molecules per AU	1	1
Matthews coefficient (Å^3^/Da)	2.70	2.73
Mosaicity (°)	0.79	0.64
Resolution range (Å)	30.88–1.67 (1.76–1.67)	41.23–1.80 (1.88–1.80)
<I/σI>	12.8 (5.9)	9.91 (2.41)
*R*_merge_ (%) *	6.1 (16.8)	7.7 (46.2)
*R*_pim_ (%) +	3.9 (11.1)	5.2 (30.9)
Multiplicity	3.0 (3.0)	2.9 (2.8)
No. of observed reflections	379108 (50315)	324367 (40070)
No. of unique reflections	124566 (16919)	108448 (9450)
Completeness (%)	85.2 (79.7)	92.3 (80.7)

* *R_merge_* = ∑*_hkl_*∑*_i_|I_i_* (*hkl*) − 〈*I*(*hkl*)〉|/∑*_hkl_*∑*_i_I_i_*(*hkl*);

+ $R_{pim}=\Sigma_{hkl}\left[\frac{1}{N-1}\right]1/2\;\Sigma_{i}|I_{i}\;(hkl)-\langle I(hkl)\rangle |\Sigma_{hkl}\;\Sigma_{i}I_{i}\;(hkl)$.

**Table 2.** SAXS Data collection and derived parameters for PaoABC.

Data collection parameters	
Instrument	EMBL X33 beam line (DORIS-III, DESY, Hamburg)
Beam geometry	2.0 × 0.6 mm^2^
Wavelength (Å)	1.5
*q* range (Å^−1^) a	0.01–0.5
Exposure time (s)	8 × 15
Concentration range (mg/mL)	0.3–20
Temperature (K)	293

Structural parameters b	

*I*(*0*) (relative) [from *p*(*r*)]	120 ± 1
*R*_g_ (Å) [from *p*(*r*)]	36 ± 1
*I*(*0*) (cm^−1^) (from Guinier)	120 ± 1
*R*_g_ (Å) (from Guinier)	35 ± 1
*D*_max_ (Å)	120 ± 10
Porod volume estimate (Å^3^)	200,000 ± 10,000
Excluded volume estimate (Å^3^)	240,000 ± 10,000

Molecular-mass determination	

*I*(*0*) (cm^−1^) BSA (66,000 Da)	60 ± 1
Molecular mass *M*_r_ (Da) [from *I*(*0*)]	130,000 ± 15,000
Molecular mass *M*_r_ (Da) [from Porod volume (*V*_p_/*1.6*)]	125,000 ± 10,000
Molecular mass *M*_r_ (Da) [from excluded volume (*V*_ex_/*2*)]	120,000 ± 10,000
Calculated monomeric *M*_r_ from sequence	136,000

Software employed	

Primary data reduction	Automated SAXS pipeline
Data processing	PRIMUS
*Ab initio* analysis	DAMMIN, DAMMIF
Validation and averaging	SUPCOMB, DAMAVER
Computation of model intensities	CRYSOL
3D graphics representations	VMD

Abbreviations: *M*_r_: molecular mass; *R*_g_: radius of gyration; *D*_max_: maximal particle dimension; *V*_p_: Porod volume; *V*_ex_: Particle excluded volume.

a Momentum transfer |*q*| = 4πsin(θ)/λ.

b Values reported for the data merged data set (0.3 & 10 mg/mL).
